# Risk Factors for Retinopathy of Prematurity in Preterm Infants in Kerman, Iran

**DOI:** 10.18502/jovr.v19i1.15437

**Published:** 2024-03-14

**Authors:** Arash Daneshtalab, Mahla Shadravan, Amirreza Mobasherzadeh Mahani, Azam Dehghani, Mahdi Sharifzadeh Kermani

**Affiliations:** ^1^Clinical Research Development Unit, Shafa Hospital, Kerman University of Medical Sciences, Kerman, Iran; ^2^Clinical Research Development Unit, Afzalipour Hospital, Kerman University of Medical Sciences, Kerman, Iran

**Keywords:** Preterm Infant, Retinopathy of Prematurity, Risk Factors

## Abstract

**Purpose:**

Retinopathy of prematurity (ROP) is the main cause of blindness in premature infants. Despite developments in neonatal care and management guidelines, ROP is becoming increasingly prevalent worldwide. Hence, the identification of risk factors for ROP is critical to diminish the burden of the disease.

**Methods:**

This cross-sectional study included all infants with gestational age ≤ 36 weeks referred to the ophthalmology clinic at Shafa hospital, Kerman, Iran from 2014 to 2015. Ophthalmologic examinations were performed followed by demographic and ophthalmic data collection and analysis.

**Results:**

A total of 579 preterm neonates were screened including 325 boys and 254 girls. The incidence of ROP was 17.96%. Mean birth weight (BW) and gestational age (GA) were 1668.0 grams and 31.2 weeks, respectively. The results indicated that BW, GA, duration of hospitalization and oxygen therapy were significantly related to the development of ROP, however, after multivariate logistic regression analysis, only BW and duration of hospitalization remained significant. There were no significant associations between gender, type of delivery, or assisted reproductive technologies, and ROP (*P* = 0.461, 0.461, and 0.826, respectively).

**Conclusion:**

BW and duration of hospitalization were significant risk factors for ROP in the current study. BW was also strongly associated with the need for therapy.

##  INTRODUCTION

Retinopathy of prematurity (ROP) is a vasoproliferative retinal disease which can lead to blindness. ROP is currently the commonest cause of avoidable blindness in low/middle-income countries.^[1,2,3]^ Along with advances in neonatal care, more cases of ROP occur in extremely low-gestational-age neonates (less than a gestational age of 28 weeks at birth) and the total number of children at risk for ROP is increasing worldwide.^[4,5]^

There is a disruption in the growth of normal vasculature in premature infants leading to aberrant angiogenesis in the retina.^[6,7]^ ROP develops in two phases: (1) inhibition of growth factors induced by hyperoxia and disruption of maternal–fetal interaction in the retinal vasculature and (2) metabolically active, yet inadequately vascularized retina becomes hypoxic, stimulating growth factor induced vasoproliferation, which can lead to retinal detachment.^[8,9]^ Therefore, understanding the ROP mechanism can contribute to the recognition of the optimal postnatal conditions in premature infants. In addition, identification of postnatal risk factors of ROP may assist neonatologists and ophthalmologists in preventing illness and limiting comorbidities.

Various risk factors including hereditary and perinatal factors, demographical and nutritional comorbidities, medical interventions, and maternal factors have been proposed for ROP.^[10,11,12,13,14]^ However, oxygen exposure, low birth weight, and low gestational age are the key risk factors of ROP.^[15]^ In some cases, factors which lead to preterm birth that may also interfere with the development of the intrauterine retinal neurovascular infrastructure. Antenatal factors including placental infection and inflammation may also make the fetal retina more susceptible to severe ROP. This heightened sensitivity could potentially trigger a precursor to the disease. ${\;}^{\left[16,17\right]}$ Additionally, fluctuations in oxygen concentrations throughout the first few weeks of the infant's life are correlated with the risk of ROP.^[18]^ A high incidence of periodic hypoxia through the first eight weeks of life often results in subsequent severe disease.^[19,20]^

Despite developments in neonatal care and administration guidelines, ROP is a common blinding disease in infants worldwide. The percentage of blindness secondary to ROP varies exceedingly among countries and is influenced by the level of neonatal care as well as the level of accessibility to powerful ROP screening and treatment plans.^[21]^ Timely screening and treatment are important to reduce the adverse outcomes including blindness.^[22]^ It is suggested that each country develops and applies its own specific screening criteria suitable for its population. Low birth weight and low gestational age are both major risk factors for ROP, however, to the best of our knowledge, no study has been conducted to differentiate between the two risk factors. Therefore, this study was designed to compare the effects of these two risk factors as well as to investigate other risk factors associated with ROP in Iran.

**Table 1:** The frequency of ROP* by gender, type of birth delivery, ART*.


	**Retinopathy**	**No- retinopathy**	**Total**	***P*** **-value**
Gender	Male	55 ± 52.9	270 ± 56.8	325 ± 56.1	0.461
	Female	49 ± 47.1	205 ± 43.2	254 ± 43.9	
Type of birth delivery	Vaginal delivery	26 ± 25	120 ± 25.3	146 ± 25.2	0.95
	Cesarean section	78 ± 75	355 ± 74.7	433 ± 74.8	
ART	Not used	89 ± 85.6	402 ± 88.09	491 ± 88.3	0.82
	IUD	1 ± 1	4 ± 0.9	5 ± 0.9	
	Ovarian activating drug	6 ± 5.8	15 ± 3.3	21 ± 3.8	
	Unclear	1 ± 1	3 ± 0.7	4 ± 0.7	
	IVF	7 ± 6.7	20 ± 4.4	27 ± 4.9	
	IU-I	0 ± 0	5 ± 1.1	5 ± 0.9	
	
	
ART, assisted reproductive technologies; ROP, retinopathy of prematurity					

**Table 2:** Key risk factors for ROP.


	**Retinopathy**	**No- retinopathy**	***P*** **-value**
Maternal age	28.33 ± 7.04	28.88 ± 6.72	0.454
Gestational age	29.88 ± 5.74	32.64 ± 5.44	0.001
Birth weight	1414 ± 476.33	1922.06 ± 570.99	0.001
Duration of hospitalization	22.17 ± 14.03	8.95 ± 9.80	0.001
Oxygen uptake time	12.74 ± 12.60	5.14 ± 7.26	0.001
	
	

**Table 3:** Determining the frequency of ROP by disease during pregnancy.


	**Retinopathy**	**No- retinopathy**	**Total**	***P*** **-value**
Nephrotic syndrome	1 ± 1	0 ± 0	1 ± 0.2	0.032
Diabetes	11 ± 10.6	50 ± 10.5	61 ± 89.5	0.988
Preeclampsia	2 ± 1.9	1 ± 0.2	3 ± 0.5	0.028
High blood pressure	16 ± 15.4	57 ± 12	73 ± 12.6	0.346
	
	

**Table 4:** Determining the frequency of ROP according to drug use during pregnancy.


	**Retinopathy**	**No-retinopathy**	**Total**	***P*** **-value**
Methyldopa	6 ± 5.8	23 ± 4.8	29 ± 5	0.695
Levothyroxine	19 ± 18.3	68 ± 14.3	87 ± 15	0.307
Insulin	8 ± 7.7	26 ± 5.5	34 ± 5.9	0.383
Aspirin	6 ± 5.8	17 ± 3.5	23 ± 3.9	0.324
	
	

**Table 5:** The result of multiple logistic regression analysis.


	***P*** **-value**	**OR**	**95% CI for OR**	
		**Lower**	**Upper**
Gestational age	0.27	1.02	0.98	1.06
Birth weight	0.001	1	1	1
Duration of hospitalization	0.001	0.93	0.91	0.96
Oxygen uptake time	0.66	0.99	0.95	1.02
Nephrotic syndrome	1	–	–	–
Preeclampsia	0.09	10.06	0.68	148.04
	
	
OR, odds ratio; CI, confidence interval				

**Table 6:** Relevance of gestational age and birth weight with required treatment.


	**Need for therapy**	**No need for therapy**	***P*** **-value**
Gestational age	30.17 ± 0.49	32.24 ± 0.24	0.056
Birth weight	1368.12 ± 78.41	1854.31 ± 24.67	0.001
	
	

**Table 7:** Stage and zone.


**Stage and Zone**	**Frequency (%)**
0&2	1 (1)
0&3	1 (1)
1&1	2 (1.9)
1&2	13 (12.5)
1&3	41 (39.4)
2&1	1 (1)
2&2	16 (5.4)
2&3	3 (2.9)
3&1	16 (15.4)
3&2	8 (7.7)
3&3	1 (1)
4&1	1 (1)
	
	

**Table 8:** Relevance of gestational age and birth weight with the stage of retinopathy of prematurity.


	**Stage 1**	**Stage 2**	**Stage 3**	***P*** **-value**
Gestational age	29.6 ± 0.86	31 ± 2.91	31.07 ± 2.36	0.33
Birth weight	1428.71 ± 78.41	1346.25 ± 100.94	1500.38 ± 86.62	0.129
	
	

##  METHODS

The current cross-sectional study was performed at the ophthalmology clinic of Shafa Hospital in Kerman, Iran from 2014 to 2015. We assessed the medical reports of the preterm infants referred to the eye clinic for ROP evaluation. Screening was carried out by trained ophthalmologists and the infants who had ROP were referred to an experienced vitreoretinal surgeon. The criteria for the screening were: gestational age ≤ 32 weeks or birth weight ≤ 2000 gr, as well as the neonates at older gestational age and higher birth weight who had one or more of the following risk factors: oxygen therapy, sepsis, blood transfusion, respiratory distress syndrome, infection. The first retinal examination of premature infants with a gestational age of ≤ 27 weeks was performed at 31 weeks of postmenstrual age. For neonates with a gestational age between 28 and 32 weeks, the first retinal examination was at 4–6 weeks after birth or postmenstrual age of 31–33 weeks (whichever was later). Demographical data (gestational age, birth weight, and gender) and ophthalmic findings for each patient were collected and categorized. Pupils were dilated with 0.5% tropicamide eye drops combined with 2.55% phenylephrine drops 1 hr before examination. Indirect ophthalmoscopy was used with an infant lid speculum and scleral indentation under topical anesthesia. Preterm infants with insufficient medical records were excluded from the study and 579 infants were selected eventually.

Data analysis was performed using the IBM SPSS version 19 (Armonk, NY: IBM Corp) software. The comparison between data of patients with and without ROP was analyzed using student *t*-test. *P*-value < 0.05 in the *t*-test was considered statistically significant. Multiple regression analysis was performed.

##  Ethical Considerations

This cross-sectional survey was approved by the Research Ethics Committee of Kerman University of Medical Sciences (Code: IR.KMU.REC.1395.366).

##  RESULTS

In this study, 579 infants including 325 males and 254 females were evaluated. The incidence of ROP was 17.96%. We observed that 55 boys and 49 girls suffered from ROP. The results of our study demonstrated that there was no significant relationship between gender and ROP (*P* = 0.461) [Table 1]. In other words, both sexes were equally likely to get the disease. The results of our study did not show a significant relationship between the method of birth delivery and the occurrence of ROP (*P* = 0.95). In other words, the ratio of neonates who suffered from ROP was identical between vaginal delivery and cesarean section. We also discovered that assisted reproductive technologies (ARTs) were not correlated to the development of ROP (*P* = 0.82) [Table 1].

Our findings revealed that birth weight, gestational age, duration of hospitalization, and oxygen uptake time were all significantly related to the occurrence of ROP (*P* = 0.001). We also assessed the association between maternal age with ROP which revealed that it was not significantly related to ROP (*P* = 0.454) [Table 2].

In addition to the aforementioned factors, the reviewed maternal medical history revealed a wide range of diseases. The reports indicated that nephrotic syndrome (*P* = 0.032) and preeclampsia in mothers correlated with ROP in their infants (*P* = 0.028); however, in the multivariate logistic regression analysis, the correlation was not statistically significant. Hypothyroidism, diabetes, and high blood pressure listed in Table 3 were also not significantly associated with ROP.

The findings of the current study revealed that there was no significant association between the occurrence of ROP in infants whose mothers took drugs listed in Table 4.

Based on multivariate logistic regression analysis, only birth weight and duration of hospitalization were statistically significant.

Our study also evaluated the correlation between gestational age and birth weight with the need for treatment in infants with ROP. It was revealed that the birth weight of an infant with ROP was strongly associated with the need for therapy (*P* = 0.001). However, such an association was not detected in the survey of the gestational age factor [Table 6].

ROP is classified based on two categories: the zone of abnormal blood vessels and the extent of abnormal vessels; dividing into 3 zones and 5 stages, respectively. Accordingly, involvement of zone 3 and stage 1 was the most frequent one (39.4%). The least frequencies (1%) were zones 2,3,1,3,1 and stages 0,0,2,3,4, respectively [Table 7].

Due to the importance of the two risk factors, gestational age and birth weight, in the occurrence of ROP, we assessed the relationship between each one at different stages of this disease. Our analysis did not present any significant relationship between the three stages of ROP and the mentioned factors [Table 8].

##  DISCUSSION

Our study revealed that birth weight, gestational age, duration of hospitalization, and oxygen uptake time were all significantly related to the occurrence of ROP. However, based on multivariate logistic regression analysis, only the birth weight and duration of hospitalization were statistically significant. Birth weight of infants with ROP was also strongly associated with the need for treatment, however, such an association was not detected in the survey of the gestational age factor; therefore, it seems that birth weight is a more influential factor compared to gestational age.

İkbal Seza and colleagues investigated the effect of birth weight on the stages of ROP in twin pairs. Since twin pairs have equal gestational age, they investigated the specific effect that birth weight may have on the development of ROP and showed that birth weight plays an important role in any stage of ROP.^[23]^ Insulin-like growth factor I (IGF-I) is an important somatic growth factor for fetal and neonates' growth and development that is correlated with birth weight.^[24,25]^ Early levels of low IGF-I are associated with slower-than-expected weight gain and more severe ROP.^[26]^ Therefore, birth weight is a more important factor than gestational age as it pertains to the development of RO.

The results of our study about the relation between gender and ROP did not show any association between these factors. Saeidi and his colleagues also conducted a study on low-birth-weight infants admitted at Imam Reza Hospital, Mashhad, Iran where their results confirmed the lack of meaningful communication between the two factors.^[27]^ In addition, several studies demonstrated the significance of maternal factors in the development of ROP such as maternal diseases, maternal drug consumption, gestational age, smoking, and white blood cell count.^[28,29,30]^ In our study, there was only one case of nephrotic syndrome in which the infant was born with ROP, two cases of preeclampsia were also reported and their infants had ROP. Although, a meta-analysis study with 13 cohort studies was conducted, no association was revealed between preeclampsia and ROP.^[31]^ The conflicting results observed in the studies could be attributed to the diverse range of study conditions and other interfering factors that were considered in the analysis. On the other hand, nephrotic syndrome has not been recorded as a risk factor in any study. Therefore, further research is recommended in this area.

Some drugs used during pregnancy are also recognized as maternal risk factors for ROP, including antihistamine, beta blockers, aspirin, non-steroidal anti-inflammatory drugs (NSAIDs), and antibiotics.^[32]^ Furthermore, in our study, using methyldopa, levothyroxine, insulin, and aspirin by mothers were not associated with the occurrence of ROP in their infants. The role of diabetes, however, has remained unclear in different societies.^[28]^

Conflicting data exists regarding the association between the method of delivery and the occurrence of ROP.^[33,34]^ Although our study did not demonstrate a significant relationship, an analysis of Italian infants suggested that vaginal delivery could pose a risk factor for infants with threshold ROP and a weight of < 1000 gr. The argument was that the mechanical pressure experienced during vaginal delivery could lead to ischemia and an imbalance in hyperoxia–hypoxia, thus serving as a risk factor for ROP. Additionally, the presence of vaginal infections was found to increase the risk of ROP.^[35]^ However, in our study, no relationship was found between the type of delivery and the risk of ROP. This disparity may be due to the composition of our study, which included all stages of ROP, while the study by Manzoni et al examined infants separately according to ROP stages.

Although multiple studies have been performed on the relationship between ART and ROP in infants, the results are still inconclusive.^[36,37]^ These studies often showed that ART such as IVF due to multiple births and low birth weight was considered as a risk factor of ROP. However, the results were challenging in other studies. Barker and colleagues assessed the prevalence of ART in multiple birth infants seen for ROP screening in a retrospective study. They found no significant difference between the number of babies developing ROP in the ART and non-ART groups which was consistent with our results.^[38]^

In summary, we identified birth weight, gestational age, duration of hospitalization, and oxygen uptake time as the most important factors that correlated with the incidence of ROP. However, based on multivariate logistic regression analysis, only the birth weight and duration of hospitalization were statistically significant. It was also revealed that the birth weight of infants with ROP was significantly associated with the need for therapy, however, such an association was not detected in the survey of the gestational age factor; therefore, it seems that birth weight is a more important risk factor as compared to gestational age.

##  Financial Support and Sponsorship

None.

##  Conflicts of Interest

None.
